# Increasing Lysine Content of *Waxy* Maize through Introgression of *Opaque-2* and *Opaque-16* Genes Using Molecular Assisted and Biochemical Development

**DOI:** 10.1371/journal.pone.0056227

**Published:** 2013-02-15

**Authors:** Wenlong Zhang, Wenpeng Yang, Mingchun Wang, Wei Wang, Guiping Zeng, Zhiwei Chen, Yilin Cai

**Affiliations:** 1 Guizhou Institute of Upland Food Crops, Guizhou Academy of Agricultural Sciences, Guiyang, Guizhou, China; 2 College of Agronomy and Biotechnology, Southwest University, Chongqing, China; 3 Guizhou General Seed Station, Guizhou Agricultural Committee, Guiyang, Guizhou, China; 4 Guizhou Key Laboratory of Agricultural Biotechnology, Guizhou Academy of Agricultural Sciences, Guiyang, Guizhou, China; 5 Agricultural College, Guizhou University, Guiyang, Guizhou, China; Cankiri Karatekin University, Turkey

## Abstract

The low lysine content of waxy maize cannot meet the nutritional requirements of humans, livestock, or poultry. In the present study, the high-lysine genes *o2* and *o16* were backcrossed into *wx* lines using the maize high-lysine inbreds TAIXI19 (*o2o2*) and QCL3021 (*o16o16*) as donors and the waxy maize inbred line QCL5019 (*wxwx*) as a receptor. In the triple-cross F_1_, backcross, and inbred generations, the SSR markers phi027 and phi112 within the *wx* and *o2* genes and the SSR marker umc1121 linked to the *o16* gene were used for foreground selection. Background selection of the whole-genome SSR markers was performed for the selected individuals. The grain lysine content was determined using the dye-binding lysine method. The waxiness of the grain was determined with the I_2_-KI staining and dual-wavelength spectrophotometric analysis. The BC_2_F_2_ generation included 7 plants of genotype *wxwxo2o2O16_*, 19 plants of genotype *wxwxo16o16O2_*, and 3 plants of genotype *wxwxo2o2o16o16*. In these seeds, the average amylopectin content was 96.67%, 96.87%, and 96.62%, respectively, which is similar to that of QCL5019. The average lysine content was 0.555%, 0.380%, and 0.616%, respectively, representing increases of 75.1%, 19.9%, 94.3%, respectively, over QCL5019. The average genetic background recovery rate of the BC_2_F_3_ families was 95.3%, 94.3%, 94.2%, respectively. Among these 3 *wxwxo2o2O16O16* families, 4 *wxwxo2o2O16o16* families, and 3 *wxwxo2o2o16o16* families, the longest imported parent donor fragment was 113.35 cM and the shortest fragment was 11.75 cM. No significant differences in lysine content were found between the BC_2_F_4_ seeds and the BC_2_F_3_ seeds in these 10 families. This allowed us to increase the lysine content of waxy corn and produce seeds with excellent nutritional characteristics suitable for human consumption, animal feed, and food processing. This may be of significance in the breeding of high-quality corn and in improvement of the nutrition of humans, livestock, and poultry.

## Introduction

Waxy maize (*Zea mays* L. sinensis Kulesh), also known as sticky maize, is one of nine sub-types of maize, first found in China and later found in other regions in Asia [1], [2]. In 1909, Collins published an accurate description of waxy maize [3]. The endosperm of the dried grain is opaque with a dull, waxy appearance. In 1922, Weatherwax found the waxy corn starch to be completely composed of branched, small-molecular-weight amylopectin [4]. In 1935, Emersonk and colleagues mapped the *wx* gene in the long arm of chromosome 9, i.e., the 59 locus close to the centromere [5]. In 1943, Sprague discovered that the maize *wx* mutant lacks amylose [6]. The major mutations in waxy maize are insertion mutation, deletion mutation, and EMS mutagenesis [7]–[10]. These mutations cause splicing errors and translation errors in pre-mRNA so that the *Wx* gene is not normally expressed. The *Wx* gene encodes granule-bound starch synthase I (GBSS-I), which determines the amylose synthesis in maize endosperm and pollen [11]. Starch in the grains of normal corn (*WxWx*) was found to be composed of amylose (25%) and amylopectin (75%). The GBSS-I activity of the *wx* mutant decreased by 5% to 95%, resulting in lower amylose content in grain and waxy corns with various levels of amylose. Meng argued that the amylose content was less than 5% in waxy maize carrying the *wx-a* gene [12]. Zhang and colleagues suggested that the presence of the *wx* gene indicated that the amylose content would be between 0 and 5%, that the *du* gene indicated that amylose content would be between 5% and 15%, and that the *ae* gene indicated the amylose content would exceed 15% [13]. Sun and colleagues suggested that *Wx* was incompletely dominant to *wx* and that a dose effect was present between the amylopectin content and the endosperm *wx* gene [14]. Liu and Li indicated that it was difficult to achieve nearly 100% of amylopectin in waxy corn [15].

The *Wx* gene was first cloned and sequenced in 1986 [16]. This gene has a single copy in the maize genome with a 3.8 kb coding sequence of 14 exons and 13 introns [17]. The start codon is located in exon 2 and the stop codon is located in exon 14. These data laid the foundation for the research and application of the *Wx* gene, including the development of molecular markers within the gene in marker-assisted selection (MAS). The MaizeGDB website has published three SSR markers for the detection of the *Wx* loci: Phi022, phi027 and phi061.

MAS can shorten the recessive gene transfer from generation to generation, accurately identify target genes, and be not subject to the influence of identification conditions and heterofertilization of the seed endosperm [18]. In recent years, MAS has been used successfully in the selection of crops resistant to insect pests and drought and in the improvement of crop quality using single gene selection, polymerization of multiple genes resistant to the same disease, polymerization of multiple genes resistant to different diseases, and polymerization of resistance genes and other genes [19]–[27].

The level and types of amino acids found in maize grain, especially essential amino acids, is an important indicator of nutritional quality [28]. Generally, the humans should take in 51 mg lysine per gram of protein [29]. This requires the lysine content be more than 0.5% in maize grain. Livestock and poultry feed must be 0.6–0.8% lysine [30]. Waxy maize has excellent taste, texture, and other culinary qualities, but its nutritional value is relatively low. A survey of 93 samples of waxy corn grown in China’s Yunnan Province found them to have a lysine content of 0.24–0.34%. A survey of 40 temperate waxy corns, showed the lysine content to be 0.14–0.39% [31]. The current *opaque-2* (*o2*) maize grain contains circa 0.4% lysine, which does not meet standards for either food or fodder. However, the gene pyramiding of the *opaque-16* (*o16*) and *o2* genes has been found to significantly increase lysine content [32], [33].

The main purpose of this study was to improve the nutritional quality of waxy corn by backcrossing the two high-lysine genes *o2* and *o16* into *wx* maize line using the multi-gene MAS combined with biochemical techniques, to produce waxy seeds with high lysine content, and to promote high-quality corn breeding and development of relevant industries.

## Materials and Methods

### Parent Materials and Population Construction

TAIXI19 is an inbred line of *o2* maize, the seed lysine content of which is about 0.43%. QCL3021 and QCL5019 are inbred lines of *o16* maize and waxy maize, the seed lysine content of which are 0.32% and 0.28%, respectively. The methods used for analysis of lysine content are as follows.

The 350 kernels seeds of three F_1_ hybrid combinations were generated using TAIXI19 as the female parent and QCL3021 as the male parent in the field. The 400 kernels seeds of two triple hybrid populations were generated using the F_1_ hybrid as the female parent and QCL5019 as the male parent in the field. In the triple hybrid F_1_ generation, the 400 kernels seeds were sown in the field and 375 plants emerged; the 83 target individual plants, double heterozygous at the *o2* and *o16* loci, were selected using foreground selection and used for backcross with recurrent parent QCL5019; 72 plants of them were harvested, and the 240 kernels seeds from the two plants, G31 and G167, were selected. In the BC_1_F_1_, the 240 kernels seeds were sowed in the field and 237 plants emerged; the 20 target plants with genotype of *wxwxO2o2O16o16* were selected using foreground selection and used for backcross with recurrent parent QCL5019; 14 plants of them were harvested, and the 220 kernels seeds from two plants, G31–101 and G167–181, were selected after background selection and quality analysis. In the BC_2_F_1_, the 220 kernels seeds were sowed in the field, and 213 plants emerged; the 41 target plants with genotype of *wxwxO2o2O16o16* were selected using foreground selection and selfed; 30 plants of them were harvested, and the 340 kernels seeds from six plants, G31-101-39, G31-101-51, G31-101-110, G31-101-130, G167-181-169, and G167-181-204, were selected after background selection and quality analysis. In the BC_2_F_2_ generations, the 340 kernels seeds were sowed in the field, and 285 plants emerged; the 232 *wxwxO2_O16_* plants, 12 *wxwxo2o2O16_* plants, 35 *wxwxo16o16O2_* plants, and 6 *wxwxo2o2o16o16* plants were selected using foreground selection and selfed; 142, 7, 19, and 3 plants were harvested from each group; and the seeds from the 7 *wxwxo2o2O16_* plants (G31-101-51-67, G31-101-110-100, G31-101-110-120, G31-101-130-134, G31-101-130-135, G167-181-169-240, G167-181-204-263) and 3 *wxwxo2o2o16o16* plants (G31-101-51-62, G31-101-110-122, G167-181-204-261) were reserved after quality analysis. In the BC_2_F_3_ generation, these 10 families were grown by row in the field and their genotypes were verified using molecular markers; background analysis was performed for the whole genome; and all of families were inbred to produce the BC_2_F_4_ seeds.

### DNA Extraction, PCR Amplification, and Electrophoresis

Young, seedling-stage leaves were collected for extraction of genomic DNA of individual plants of parents and each generation using the CTAB method for corn MAS [34]. PCR amplification and electrophoresis detection of amplification products was performed as reported previously [32], [35]. PCR amplification was performed using a 2720 Thermal Cycler (Applied Biosystems, Foster City, CA, USA) and a DNA Engine Peltier Thermal Cycler (Bio-Rad, Hercules, CA, USA). Amplification products were separated using a Sequi-Gen® GT DNA electrophoresis system (Bio-Rad).

### Seed Lysine and Starch Content Measurement

Seed lysine content was measured using acid orange-12 dye-binding lysine colorimetry (DBL) [35]. Each sample was measured 2 or 3 times and the measurements were averaged. Seed waxiness was qualitatively and quantitatively determined using I_2_-KI staining and dual wavelength spectrophotometry (DWLS), respectively [36], [37]. For quantitative determination, the absorption spectra of amylose and amylopectin were scanned using SPECORD 40 (Analytik Jena AG, Jena, Germany). Three repeated measurements were performed and averaged.

### Foreground Selection and Background Selection

#### Foreground selection (FS)

FS refers to selection of the target genes *o2*, *o16*, and *wx*. The *o2* gene was detected using the SSR markers phil12, umc1066, and phi057 within the gene [32]. The *o16* gene was detected using the linked SSR markers umc1141 and umc1121 [32]. The *wx* gene was detected using the SSR markers phi022, phi027 and phi061 [38].

#### Background selection (BS)

BS refers to selection of the genetic background of the FS-selected individuals. Parental polymorphic SSR markers from genome-wide screening were used for BS. Polymorphic markers in the BS were divided into two categories. The first was a class of markers found to be polymorphic among the three parents. The second was a class of markers found to be polymorphic between the recurrent parent and the other donor parents but not between the two donor parents.

The PCR amplification primer sequences for the SSR markers in FS and BS were adopted from the Maizegdb website (http://www.maizegdb.org) and synthesized by Shanghai Generay Biotech Co., Ltd (Shanghai, China).

### Statistical Analysis

Electrophoresis band patterns A, B, H, and U of the SSR markers were used to establish the database. In the same migration position, a band pattern consistent with the recurrent parent was recorded as A, while a band pattern consistent with the donor parent was recorded as B. A heterozygous band pattern was recorded as H and an unidentified band pattern was recorded as U. Based on the statistical analysis of genetic background recovery rate of molecular markers, the formula G (g) = [L+X (g)]/(2L) was used to calculate the background recovery rate of the FS-selected individuals after BS. Here, G (g) indicates the genetic background recovery rate in the backcross g-generation, X (g) the number of molecular markers with the band pattern of receptor parent in the backcross g-generation, and L the number of molecular markers included in the analysis [39]–[41]. The theoretical genetic background recovery rate was calculated using the formula E [G (g)] = 1−(1/2)^g+1^, where g refers to the number of backcross generations.

Analysis of variance and calculation of standard deviation were performed using SPSS13.0 software. The absorption spectra of amylose and amylopectin were plotted using Origin7.5 software. Graphical genotypes were analyzed and illustrated using GGT32 software with reference to an IBM2 2008 Neighbors Map.

## Results

### Polymorphism of SSR Markers at Target Loci and Whole Genome between the Parents

**Figure 1:**
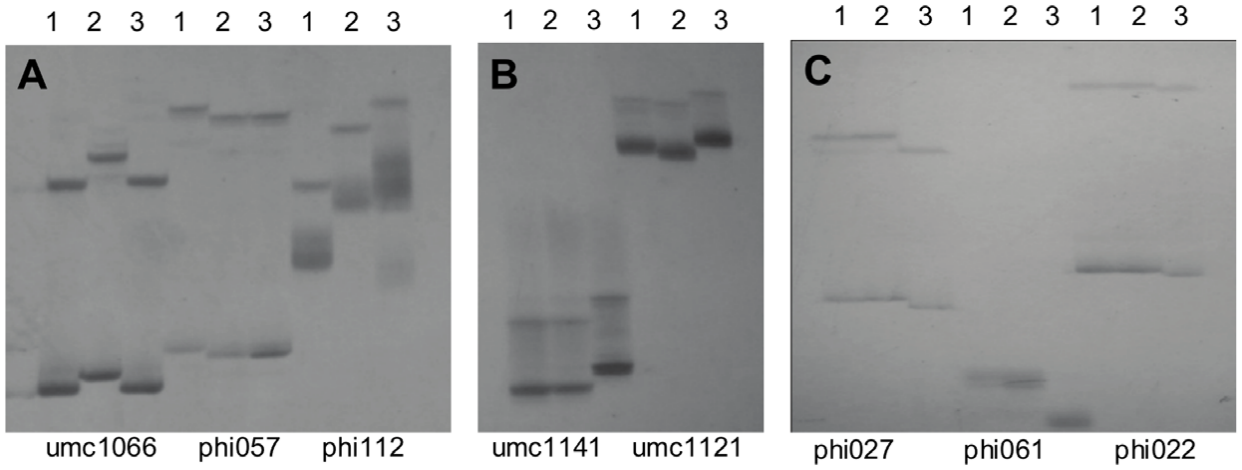
Electrophoresis pattern of SSR markers among three parents for target genes. (A) pattern of SSR markers within *opaque-2* gene; (B) pattern of SSR markers linked to *opaque-16* gene; (C) pattern of SSR markers within *waxy* gene; 1, Taixi19; 2, QCL3021; 3, QCL5019.

As shown in Figure 1, among 3 markers (umc1066, phi057, and phil12) of the *o2* gene, two marker loci (phi057, and phil12) were found to be polymorphic between TAIXI19 and the other two parents. Among the two markers in the *o16* gene, the umc1121 locus showed polymorphism between QCL3021 with the other two parents. All 3 markers (phi027, phi061, and phi022) of the *wx* gene showed polymorphism between QCL5019 and the other two parents. These polymorphic markers were codominant, which rendered them usable for the MAS of the corresponding target genes. In the present study, the markers phil12, umc1121, and phi027 were selected for FS.

Two hundred and sixty-six SSR markers distributed on the 10 chromosomes of the maize genome were selected for the screening of polymorphisms between the three parents. Of these markers, 49 were found to be polymorphic between QCL5019 and the other two parents, and 33 markers were found to be polymorphic among the three parents. A total of 82 markers were used for BS, with an overall polymorphism ratio of 30.8% (Table 1).

**Table 1:** Polymorphic SSR markers screened among QCL5019, Taixi19, and QCL3021 through whole genome in maize.

Chr.	Bin	Number of markers	Polymorphic markers	Chr.	Bin	Number of markers	Polymorphic markers
1	1.00–1.12	31	8	6	6.00–6.08	23	10
2	2.00–2.10	22	5	**7**	7.00–7.06	23	12
3	3.00–3.10	24	3	8	8.01–8.09	25	9
4	4.00–4.11	24	8	9	9.00–9.07	25	9
5	5.00–5.09	23	6	10	10.00–10.07	23	9

### Foreground Selection of the Target Genes in Various Segregating Generations

Because phil12 and phi027 served as markers within the target gene and QCL5019 was the recurrent parent, in every segregating generation of the triple-cross F_1_, BC_1_F_1_, and BC_2_F_1_, the *wx* locus of every individual was detected first, followed by the *o2* locus of *wx*-selected individuals and the *o16* locus of individuals selected from the *wx* and *o2* loci. There were 83, 20, and 41 FS-selected individuals in the triple-cross F_1_, BC_1_F_1_, and BC_2_F_1_ generations, and 72, 14 and 30 plants were harvested from each group (Table 2).

**Table 2:** Foreground selection for 3 segregating population using SSR markers.

		Phi027 (*wx*)		Phi112 (*o2*)		Umc1121 (*o16*)		
Generation	Number of plants	*Wxwx*	*wxwx*	*O2O2*	*O2o2*	*O16O16*	*O16o16*	Harvested
Three-way cross F_1_	375	–	–	198	177	94	83	72
BC_1_F_1_	237	115	122	75	47	27	20	14
BC_2_F_1_	213	–	211	119	92	51	41	30

In the BC_2_F_2_ generation, seeds from 6 outstanding BC_2_F_1_ plants were selected for planting, and FS was performed for the three target loci. Among 285 individuals, 12 were selected from the *wxwxo2o2O16_* genotype, 35 from the *wxwxo16o16O2_*genotype, and 6 from the *wxwxo2o2o16o16* genotype. As shown in Table 3, seven, nineteen, and three plants were harvested from these three groups. In the BC_2_F_3_ generation, the double-recessive and triple-recessive gene pyramiding families obtained from the last generation were planted continuously. One row was planted for each family, and molecular markers at the *wx*, *o2*, and *o16* gene loci were detected and verified. Finally, in the BC_2_F_4_ generation, 3 *wxwxo2o2O16O16* families, 4 *wxwxo2o2O16o16* families, and 3 *wxwxo2o2o16o16* families were produced.

**Table 3:** Foreground selection for BC_2_F_2_ population using SSR markers.

		Plants with one recessive gene	Plants with two recessive genes		Plants with three recessive genes
BC_2_F_1_ plantnumber	Number ofBC_2_F_2_ plants	*wxwxO2_O16_*	*wxwxo2o2O16_*	*wxwxo16o16O2_*	*wxwxo2o2o16o16*
39	59	49	2	7	1
51	16	6	3	6	1
110	55	42	2	9	2
130	58	54	3	1	0
169	59	52	1	5	1
204	38	29	1	7	1
Total	285	232	12	35	6
Harvested	171	142	7	19	3

### Selection of Genetic Background Molecular Markers in Various Segregating Generations

In the BC_1_F_1_ generation, the genetic background recovery rate of selected individuals was 73.8–86.6% with an average of 81.8%. This was 6.8% higher than the theoretical value. Two individuals with a recovery rate of 84.8% were selected for backcrossing. In the BC_2_F_1_ generation, the genetic background recovery rate of selected individuals was 85.9–92.7%, with an average of 90.42%. This was 2.92% higher than the theoretical value. The genetic background recovery rate of all the six plants selected from the BC_2_F_1_ generation was higher than 87.5%. The genetic background selection was not conducted in the BC_2_F_2_, and all the seven *wxwxo2o2O16_* plants and the three *wxwxo2o2o16o16* plants were chosen. The genetic background recovery rate of the 10 preferred families in the BC_2_F_3_ generation ranged from 93.4% to 96.3% (Table 4).

**Table 4:** Background analysis for 10 selected families in BC_2_F_3_.

BC_2_F_3_familiynumber	Recoveryrate (%)	Donor parentgenome (%)	Heterozygotegenome (%)	Unidentifiedgenome (%)	BC_2_F_3_ family number	Recoveryrate (%)	Donor parentgenome (%)	Heterozygotegenome (%)	Unidentifiedgenome (%)
62	94.6	4.2	0.6	0.6	134	95.2	3.0	1.8	0
67	96.3	1.8	1.9	0	135	94.6	2.4	2.4	0.6
100	94.6	3.6	1.2	0.6	240	95.8	3.0	0	1.2
120	96.3	1.8	1.9	0	261	94.6	3.0	1.2	1.2
122	93.4	4.8	0.6	1.2	263	94.0	4.2	1.2	0.6

The amount of donor parent genome in the 10 families of the BC_2_F_3_ generation was between 1.8% and 4.8%. The amount of heterozygote genome was 0–2.4%, and the amount of unidentified genome was 0–1.2%. Among the 10 families, families 67 and 120 had the highest genetic background recovery rate, and family 122 had the lowest recovery rate (Table 4). Family 122 also carried the longest donor fragment, which was 535.4 cM in length, and family 67 carried the shortest donor fragment, which was 200.75 cM in length. Among all families, the longest fragment imported from the donor parents was 113.35 cM, and it was located on chromosome 3. The shortest fragment imported from the donor parents was 11.75 cM, and it was located on chromosome 1.

### Graphical Genotype Analysis of the Chromosome of the Target Gene

**Figure 2:**
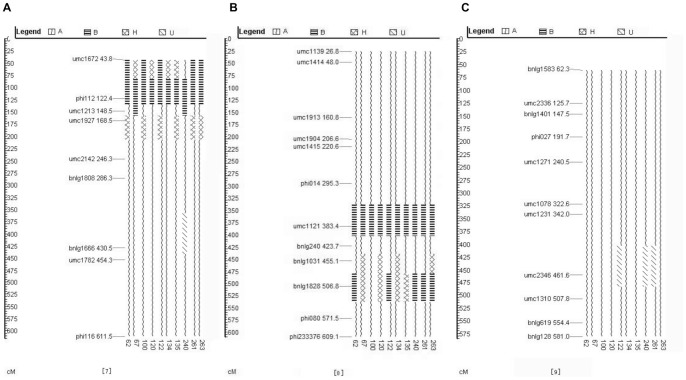
Graphical genotypes of 10 selected family lines in BC_2_F_3_ on chromosomes 7, 8, and 9. (A) graphical genotype on chromosome 7; (B) graphical genotype on chromosome 8; (C) graphical genotype on chromosome 9.

The genetic background recovery rate on chromosome 7 in 10 families of the BC_2_F_3_ generation was 87.5–93.8%. The relative amount of donor parent fragment was 0–6.25%, and the proportion of heterozygous fragment was 0–12.5%. The genetic background recovery rate on chromosome 8 was 90.9–95.5%. The proportion of the donor parent fragment was 0–4.5%, and the proportion of heterozygous fragment was 0–9.1%. The genetic background recovery rate on chromosome 9 was 95.0–100%. Except the individuals of 3 families (122, 240, and 261) had an unidentified fragment, the recovery rate of all the other individuals approached 100% (Figure 2).

Family 240 had the shortest foreign fragment imported on chromosome 7, families 120, 134, and 135 shared the shortest donor parent fragment, and family 240 had no imported heterozygous fragment. Family 100 had the shortest foreign fragment imported on chromosome 8, families 67, 100, 120, 134, and 135 had the shortest donor parent fragment, and families 62, 100, 122, 240, and 261 had no imported heterozygous fragment. Any imported foreign fragment was not found on chromosome 9, only 3 families contained an unidentified fragment (Figure 2).

### Analysis of the Donor Allele in Ten Preferred Families

The imported donor parent genomes of 10 preferred families were divided into three types: B1 - consistent with the alleles of donor parent TAIXI19; B2 - consistent with the alleles of donor parent QCL3021; and B3 - consistent with the alleles of both donor parents. B1 made up 0.6–2.4% of the total, B2 0–1.8%, and B3 0–1.2% (Table 5).

**Table 5:** Proportion of donor parent allele of 10 selected families in BC_2_F_3_.

BC_2_F_3_ family number	Allele of Taixi19 (%)	Allele of QCL3021 (%)	Allele of Taixi19 and QCL3021 (%)
62	1.2	1.8	1.2
67	0.6	0.6	0.6
100	1.2	1.2	1.2
120	1.2	0	0.6
122	1.8	1.8	1.2
134	2.4	0	0.6
135	1.8	0.6	0
240	1.8	0.6	0.6
261	0.6	1.8	0.6
263	1.2	1.8	1.2

The imported donor fragments distributed on chromosomes 1, 3, 4, 6, 7, and 8 contained the following 10 loci: umc1124 (1), umc1619 (1), umc2100 (1), umc2025 (1), phi053 (3), umc2188 (4), mc1014 (6), umc1672 (7), umc1213 (7), and bnlg1828 (8). Except for umc1619 and umc1672, which both had polymorphisms between recurrent parent and two donor parents, all 8 marker loci had polymorphisms between the three parents.

### Lysine Content in Various Generations

The lysine content was 0.261–0.337% in 72 seeds of the BC_1_F_1_ generation, 0.278–0.362% in 14 seeds of the BC_2_F_1_ generation, and 0.346–0.549% in 30 seeds of the BC_2_F_2_ generation. In the BC_2_F_3_ generation, 171 seeds were harvested and 169 seeds were measured for lysine content (see discussion section for usage of the other 2 seeds). Analysis of variance showed that the lysine content was significantly different between different genotypes (*P*<0.01). These were, from highest to lowest, *wxwxo2o2o16o16*> *wxwxo2o2O16_*>*wxwxO2_o16o16*> *wxwxO2_O16_*. They had an average lysine content of 0.616%, 0.555%, 0.323%, and 0.282% respectively. These values were 94.3%, 75.1%, and 19.9% higher, respectively, than those of *wxwx* parent (Table 6). This indicates that the triple-recessive gene pyramiding families and double-recessive gene pyramiding families have significant positive interaction effects and that the regulatory role of the *o2* gene is greater than that of the *o16* gene. The lysine content ranged from 0.505% to 0.639% in 3 *wxwxo16o16o2o2* families and 7 *wxwxO16_o2o2* families; 59.3–101.6% higher than the recurrent parent; 8.1–36.8% higher than the high-value parent *o2* line; and 38.7–75.5% higher than the low-value parent *o16* line (Table 7). No significant difference in lysine content was found between BC_2_F_4_ seeds and BC_2_F_3_ seeds (*P*>0.05) (Table 7 and 8), suggesting that the lysine content tends to stabilize.

**Table 6:** Lysine content of BC_2_F_3_ seeds from four recessive genotypes in BC_2_F_2_.

BC_2_F_2_ plant genotype	Number ofsamples	Range oflysine (%)	Lysine content (Mean ± SD) (%)	Difference from“*o2o2”* (%)^a^	Difference from “*o16o16”* (%)^b^	Difference from “*wxwx*” (%)^c^
*wxwxo16o16o2o2*	3	0.589–0.639	0.616±0.025	31.9	69.2	94.3
*wxwxO16_o2o2*	7	0.505–0.592	0.555±0.028	18.8	52.4	75.1
*wxwxo16o16O2_*	17	0.323–0.404	0.380±0.021	/	4.4	19.9
*wxwxO16_ O2_*	142	0.282–0.367	0.332±0.024	/	/	/

a,b,c :Differences from “*o2o2,*” “*o16o16,*” and “*wxwx*” (%) indicate the average lysine content of each recessive genotype relative to the parent lines. Values given are relative increases and decreases.

**Table 7:** Lysine content of 10 selected families BC_2_F_3_ seeds and their parental seeds.

BC_2_F_3_ family number	Genotype^a^	Lysine content(Mean ± SD) (%)	Difference from“*o2o2”* (%)^b^	Difference from “*o16o16”* (%)^c^	Difference from“*wxwx”* (%)^d^
62	*wxwxo16o16o2o2*	0.589±0.020	26.1	61.8	85.8
67	*wxwxO16O16o2o2*	0.505±0.009	8.1	38.7	59.3
100	*wxwxO16o16o2o2*	0.592±0.011	26.8	62.6	86.8
120	*wxwxO16O16o2o2*	0.537±0.006	15.0	47.5	69.4
122	*wxwxo16o16o2o2*	0.639±0.011	36.8	75.5	101.6
134	*wxwxO16o16o2o2*	0.555±0.008	18.8	52.5	75.1
135	*wxwxO16O16o2o2*	0.558±0.014	19.5	53.3	76.0
240	*wxwxO16o16o2o2*	0.573±0.017	22.7	57.4	80.8
261	*wxwxo16o16o2o2*	0.621±0.012	33.0	70.6	95.9
263	*wxwxO16o16o2o2*	0.566±0.009	21.2	55.5	78.5
Taixi19	*WxWxO16O16o2o2*	0.467±0.019	/	28.3	47.3
QCL3021	*WxWxo16o16O2O2*	0.364±0.010	−22.1	/	14.8
QCL5019	*wxwxO16O16O2O2*	0.317±0.022	−32.1	−12.9	/
Normal hybrid	*WxWxO16O16O2O2*	0.265±0.014	−43.3	−27.2	−16.4

a :Genotypes containing *o2*, *o16*, and *wx* loci were validated using phi112, umc1121, and phi027 markers. “*o2o2*” is the genotype of Taixi 19, “*o16o16*” is the genotype of QCL3021, and “*wxwx*” is the genotype of QCL5019.

b,c,d :changes relative to “*o2o2,*” “*o16o16,*” and “*wxwx*” (%) indicate the average lysine content of each recessive genotype relative to the parent lines. Values are given in relative increases and decreases.

**Table 8:** Lysine content of 10 selected families BC_2_F_4_ seeds and their parental seeds.

**BC_2_F_4_ family number**	**Genotype** a	**Lysine content** **(Mean ± SD) (%)**	**Difference from** **“** ***o2o2*** **” (%)^b^**	**Difference from “** ***o16o16*** **” (%)^c^**	**Difference from** **“** ***wxwx*** **” (%)^d^**
62	*wxwxo16o16o2o2*	0.600±0.012	27.1	60.9	85.1
67	*wxwxO16O16o2o2*	0.525±0.013	11.2	40.8	62.0
100	*wxwxO16o16o2o2*	0.582±0.015	23.3	56.0	79.6
120	*wxwxO16O16o2o2*	0.562±0.137	19.1	50.7	73.5
122	*wxwxo16o16o2o2*	0.660±0.016	39.8	76.9	103.7
134	*wxwxO16o16o2o2*	0.568±0.013	20.3	52.3	75.3
135	*wxwxO16O16o2o2*	0.544±0.014	15.3	45.8	67.9
240	*wxwxO16o16o2o2*	0.577±0.023	22.2	54.7	78.1
261	*wxwxo16o16o2o2*	0.615±0.026	30.3	64.9	89.8
263	*wxwxO16o16o2o2*	0.576±0.009	22.0	54.4	77.8
Taixi19	*WxWxO16O16o2o2*	0.472±0.022	/	26.5	45.7
QCL3021	*WxWxo16o16O2O2*	0.373±0.037	−21.0	/	15.1
QCL5019	*wxwxO16O16O2O2*	0.324±0.017	−31.4	−13.1	/
Normal hybrid	*WxWxO16O16O2O2*	0.288±0.046	−40.0	−22.8	−11.1

a :Genotypes containing *o2*, *o16*, and *wx* loci were validated using phi112, umc1121, and phi027 markers. “*o2o2*” is the genotype of Taixi 19, “*o16o16*” is the genotype of QCL3021, and “*wxwx*” is the genotype of QCL5019.

b,c,d :changes relative to “*o2o2,*” “*o16o16,*” and “*wxwx*” (%) indicate the average lysine content of each recessive genotype relative to the parent lines. Values are given in relative increases and decreases.

### Qualitative and Quantitative Determination of Starch Content in Various Generations

The BC_1_F_1_, BC_2_F_1_, and BC_2_F_2_ seeds selected by FS from the triple-cross F_1_, BC_1_F_1_, and BC_2_F_1_ generations were qualitatively identified using I_2_-KI staining. Seeds whose endosperms were stained umber were selected. The DWLS method was used to quantitatively determine the levels of amylose and amylopectin in seeds of the 10 selected families. Amylopectin made up 96.26–97.06% of the total starch content. This is similar to the 96.84% observed in QCL5019. The total starch content was 53.94–56.17%, which was lower than 67.62% in QCL5019 (*P*<0.01) (Table 9).

**Table 9:** Starch and amylopectin content of 10 selected families BC_2_F_3_ seeds.

BC_2_F_3_ family number	Genotype	Total starch content (mean ± SD) (%)	Amylopectin content (mean ± SD) (%)
62	*wxwxo16o16o2o2*	55.21±0.55	97.06±0.05
67	*wxwxO16O16o2o2*	54.87±0.29	96.77±0.08
100	*wxwxO16o16o2o2*	55.62±0.16	96.45±0.04
120	*wxwxO16O16o2o2*	56.17±0.11	96.61±0.12
122	*wxwxo16o16o2o2*	54.44±0.39	96.99±0.26
134	*wxwxO16o16o2o2*	54.67±0.73	96.45±0.05
135	*wxwxO16O16o2o2*	55.55±0.19	96.37±0.07
240	*wxwxO16o16o2o2*	54.19±0.14	96.74±0.06
261	*wxwxo16o16o2o2*	54.86±0.48	96.85±0.06
263	*wxwxO16o16o2o2*	53.94±0.40	96.26±0.05
QCL5019	*wxwxO16O16O2O2*	67.62±0.68	96.84±0.20

## Discussion

In the present study, MAS technology was used to produce 3 *wxwxo2o2o16o16* families. In these families, average lysine content was found to reach 0.616% and the amylopectin content was found to reach 96.62%. The lysine content in these seeds, which tend to be waxy, has met the needs of people, livestock, and poultry. These seeds are of some importance in the genetic improvement and breeding of special types of corn.

Recessive genes have their own specific genetic effects [42]. The interactions within the double-recessive or triple-recessive mutations formed by gene pyramiding can affect the quantity and quality of starch, sugar, and protein in the endosperm [43]. This can affect seed emergence, seedling growth, and flowering time agreement, causing a low level of FS selection and a reduced harvest after pollination. This increases the difficulty of selection and necessitates a large population for selection. In the present study, 340 kernels of seeds from 6 families were planted in the BC_2_F_2_ generation. Of these seeds, 285 emerged. Then 6 *wxwxo2o2o16o16* plants, 12 *wxwxo2o2O16_* plants, and 35 *wxwxo16o16O2_* plants were selected using FS. Finally, and 3, 7, and 19 plants were harvested from each of the three genotypic families.

Li and Liu argued that the double-recessive mutations formed by *o2* and *wx* were not associated with significant changes in the total starch content of the grain [44]. However, in the present study, the total starch content of the seeds containing the *o2* gene with double-recessive (*wxwxO16_o2o2*) and triple-recessive (*wxwxo16o16o2o2*) mutations were 55.22% and 54.33%, respectively, significantly less than the recurrent parent QCL5019 (67.62%, *P*<0.01). This may be related to the differences in genetic background or to the hybrid model.

**Figure 3:**
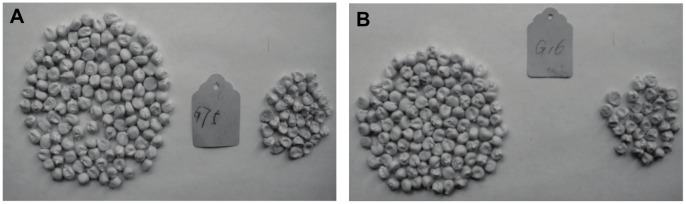
Smooth and shrunken seeds of 2 family lines in BC_2_F_3_. (A) phenotype of BC_2_F_3_ seeds from BC_2_F_2_ plant with the genotype *wxwxo16o16O2o2* (G75); (B) phenotype of BC_2_F_3_ seeds from BC_2_F_2_ plant with the genotype *wxwxo16o16O2o2* (G16).

In the present study, the BC_2_F_3_ seeds of BC_2_F_2_ plants with genotypes *wxwxO16_ O2_* and *wxwxo16o16O2_* were plump and smooth, but the BC_2_F_3_ seeds of BC_2_F_2_ plants with genotypes *wxwxO16_o2o2* and *wxwxo16o16o2o2* were depressed and wrinkled. Two BC_2_F_3_ seeds from BC_2_F_2_ plants with the genotype *wxwxo16o16O2o2* were selected from 19 BC_2_F_3_ seeds from the BC_2_F_2_ generation with the genotype *wxwxo16o16O2_* (No. G75 and G16, Figure 3). Of these, 240 seeds were smooth and 70 seeds were wrinkled, with an indoor germination emergence of 205 and 45 plants, respectively. Using phi112 detection, the *wxwxo16o16O2o2* and *wxwxo16o16O2O2* genotypes accounted for 97.6% of all smooth seeds, and 100% of shrunken seeds had the *wxwxo16o16o2o2* genotype. The same detection process was applied to two BC_2_F_4_ seeds from BC_2_F_3_ plants with the genotype *wxwxo16o16O2o2*. Results showed the phenotype and genotype concordance rates to be 97.1% and 100%, respectively (Table 10). These findings indicate that the interactions between the *o2* and *wx* genes in the endosperm cause the grain endosperm to shrink. The BC_2_F_1_ seeds were obtained through a backcross with plants from the BC_1_F_1_ generation of the genotype *wxwxO16o16O2o2*. Pale yellow seeds with high lysine content (G89) were phenotypically selected for three seasons of continuous self-breeding and the pyramiding yellow grain was harvested. The genotypes of these yellow grains were the same as those of the above listed white grains. In this way, the goal of selection was reached through marker-assisted selection of the early generations combined with phenotypic selection of the subsequent generations. This may also reduce the cost of experiments.

**Table 10:** Phenotypes of 4 families seeds used for identifying relationships between phenotype and genotype.

Generation	BC_2_F_3_				BC_2_F_4_			
Family number	G75		G16		W47		W54	
Phenotype	Smooth	Shrunken	Smooth	Shrunken	Smooth	Shrunken	Smooth	Shrunken
Kernel	128	37	112	33	31	12	46	18
Seeding	106	25	99	20	28	6	41	7
Difference^a^	3	0	2	0	0	0	2	0
Coincidence rate (%)	97.2	100	98.0	100	100	100	95.1	100

a Number of different kernels between phenotype and genotype.

**Figure 4:**
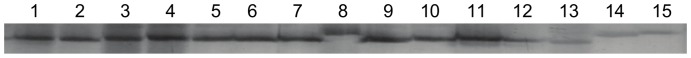
Electrophoresis pattern of 15 materials at SSR marker umc1216 locus of an endosperm modifier. 1, Taixi 19; 2, QCL 3021; 3, QCL 5019; 4, CML171; 5, (Taixi19×QCL 3021)F_1_; 6, (QCL 5019×QCL3021)F_1_; 7, (QCL 5019×Taixi 19)F_1_; 8, Nanbeinuo; 9, G122; 10, G261; 11, G67; 12, G134; 13, CML162; 14, CML193; 15, QCL2179.

The endosperm of the *o2* mutant is soft, fragile, rich in water, and readily susceptible to disease. The endosperm modifier gene can change soft endosperm into hard endosperm, and so mitigate these and other weaknesses. We used the modifier gene SSR marker umc1216 to detect the following 15 materials: TAIXI19 (*o2o2*), QCL3021 (*o16o16*), QCL5019 (*wxwx*), CML171 (modified *o2o2*), (TAIXI19 × QCL3021) F_1_, (QCL5019 × QCL3021) F_1_, (QCL5019 × TAIXI19) F_1_, NANBEINUO (*wxwx*), G122 (*wxwxo16o16o2o2*), G261 (*wxwxo16o16o2o2*), G67 (*wxwxO16O16o2o2*), G134 (*wxwxO16o16o2o2*), CML162 (modified *o2o2*), CML193 (modified *o2o2*), and QCL2179 (Normal). Our results showed that TAIXI19, QCL3021, QCL5019, CML171, (TAIXI19 × QCL3021) F_1_, (QCL5 019 × QCL3021) F_1_, (QCL5019 × TAIXI19) F_1_, G122, G261, G67, G134, and CML162 had the same allele of the modifier gene, but NANBEINUO, CML193, and QCL2179, had another allele (Figure 4). This indicates that the *wxwxo16o16o2o2* and *wxwxO16_o2o2* families have excellent endosperm texture.

In the 10 preferred families, except that 4 *wxwxo2o2O16o16* families (G100, G134, G240 and G263) need to be purified at *O16* locus, 3 *wxwxo2o2O16O16* families (G62, G122 and G261) and 3 *wxwxo2o2o16o16* families (G67, G120 and G135) can be directly used in breeding programs because their recurrent parent QCL5019 has good combining ability, and used for pyramiding more other good traits.
